# Mammographic features and risk of breast cancer death among women with invasive screen-detected cancer in BreastScreen Norway 1996–2020

**DOI:** 10.1007/s00330-023-10369-w

**Published:** 2023-11-08

**Authors:** Nataliia Moshina, Heinrich A. Backmann, Per Skaane, Solveig Hofvind

**Affiliations:** 1 Section for breast cancer screening, Cancer Registry of Norway, Oslo, Norway; 2 Department of Radiology, Nordland Hospital Trust, Bodø, Norway; 3 Division of Radiology and Nuclear Medicine, Oslo University Hospital, Oslo, Norway; 4 Department of Health and Care Sciences, The Arctic University of Norway, Tromsø, Norway

**Keywords:** Female, Middle aged, Breast neoplasms, Mammography, Screening

## Abstract

**Objectives:**

We explored associations between mammographic features and risk of breast cancer death among women with small (<15 mm) and large (≥15 mm) invasive screen-detected breast cancer.

**Methods:**

We included data from 17,614 women diagnosed with invasive breast cancer as a result of participation in BreastScreen Norway, 1996–2020. Data on mammographic features (mass, spiculated mass, architectural distortion, asymmetric density, density with calcification and calcification alone), tumour diameter and cause of death was obtained from the Cancer Registry of Norway. Cox regression was used to estimate hazard ratios (HR) with 95% confidence intervals (CI) for breast cancer death by mammographic features using spiculated mass as reference, adjusting for age, tumour diameter and lymph node status. All analyses were dichotomised by tumour diameter (small versus large).

**Results:**

Mean age at diagnosis was 60.8 (standard deviation, SD=5.8) for 10,160 women with small tumours and 60.0 (SD=5.8) years for 7454 women with large tumours. The number of breast cancer deaths was 299 and 634, respectively. Mean time from diagnosis to death was 8.7 (SD=5.0) years for women with small tumours and 7.2 (4.6) years for women with large tumours. Using spiculated mass as reference, adjusted HR for breast cancer death among women with small tumours was 2.48 (95% CI 1.67–3.68) for calcification alone, while HR for women with large tumours was 1.30 (95% CI 1.02–1.66) for density with calcification.

**Conclusions:**

Small screen-detected invasive cancers presenting as calcification and large screen-detected cancers presenting as density with calcification were associated with the highest risk of breast cancer death.

**Clinical relevance statement:**

Small tumours (<15 mm) presented as calcification alone and large tumours (≥ 15 mm) presented as density with calcification were associated with the highest risk of breast cancer death among women with screen-detected invasive breast cancer diagnosed 1996–2020.

**Key Points:**

*• Women diagnosed with invasive screen-detected breast cancer 1996–2020 were analysed.*

*• Small screen-detected cancers presenting as calcification alone resulted in the highest risk of breast cancer death.*

*• Large screen-detected cancers presenting as density with calcification resulted in the highest risk of breast cancer death.*

**Supplementary Information:**

The online version contains supplementary material available at 10.1007/s00330-023-10369-w.

## Introduction

Breast cancer survival is highly determined by histopathologic tumour characteristics at diagnosis as they define prognosis of the disease [1, 2]. Mammographic features of breast tumours [3, 4] have been shown to correlate with histopathologic tumour characteristics and linked to breast cancer survival [5–8]. The association has drawn specific attention in screening programs where the tumour diameter is known to be smaller than for symptomatic breast cancer [6, 9–15]. Studies have reported stellate or spiculated features to be prognostically favourable, while casting calcifications to be less favourable for women with breast tumours <15 mm [6, 10–12, 16].

To the best of our knowledge, the evidence on associations between mammographic features, histopathologic characteristics and disease specific survival is yet limited [6, 8–14, 16, 17]. A study from Sweden with 24 years of follow-up reported poorer breast cancer survival for women with casting type calcifications for tumours <15 mm compared to women without casting calcifications [6]. To increase radiologists’ attention to the tumours on screening mammograms associated with reduced breast cancer survival, more knowledge is needed. Furthermore, mammographic density linked to masking effect should also be considered in assessment of mammographic features and their association with prognosis [7, 18, 19].

To contribute to filling the knowledge gaps described above, we took advantage of the data collected in BreastScreen Norway since 1996 and explored the association between mammographic features and risk of breast cancer death among women with small (<15 mm in diameter) and large (≥15 mm in diameter) invasive screen-detected breast cancer. Based on previous studies, we hypothesised that calcification would be associated with the highest risk of breast cancer death for small tumours, while density with calcifications would be associated with the highest risk of breast cancer death for women with large tumours [5, 6, 11, 12, 20].

## Materials and methods

The study was reviewed by the data protection officer for research at Oslo University Hospital (PVO 20/12601).

### Study design and participants

This retrospective study was based on information from BreastScreen Norway, offering all women aged 50–69 two-view mammographic screening, biennially [21]. However, due to the biennial screening interval, the screening participants’ age range was 50–71 years. The women are invited to screening in birth cohorts associated with their place of residence, resulting in invitation of women aged 48–51 to their first examination and therefore women aged 69–72 to their last examination in the program. The program started in 1996, became nationwide in 2005 and targeted about 650,000 women in 2022. The participation rate was 76% (234,717/321,313) in 2021, and the rate of invasive screen-detected cancer 5.2 (1215/234,717) per 1000 screening examinations [21]. All screening mammograms are independently read by two breast radiologists, while mammograms with suspicious findings indicated by at least one radiologist are discussed in a consensus meeting where it is decided whether the women should be recalled [5, 17, 21]. Recalls and work-up, including supplemental imaging and biopsies, take place at dedicated breast units [21]. Mammographic features of recalls are classified by the radiologists, using a modified Breast Imaging-Reporting and Data System (BI-RADS) system [3, 4, 17]. All cancer cases are reported to the Cancer Registry. The reporting system is mandated by a law, set in 1952, and the Cancer Registry is thus considered almost complete for solid malignant tumours [22].

### Variables and data measurement

**Fig. 1:**
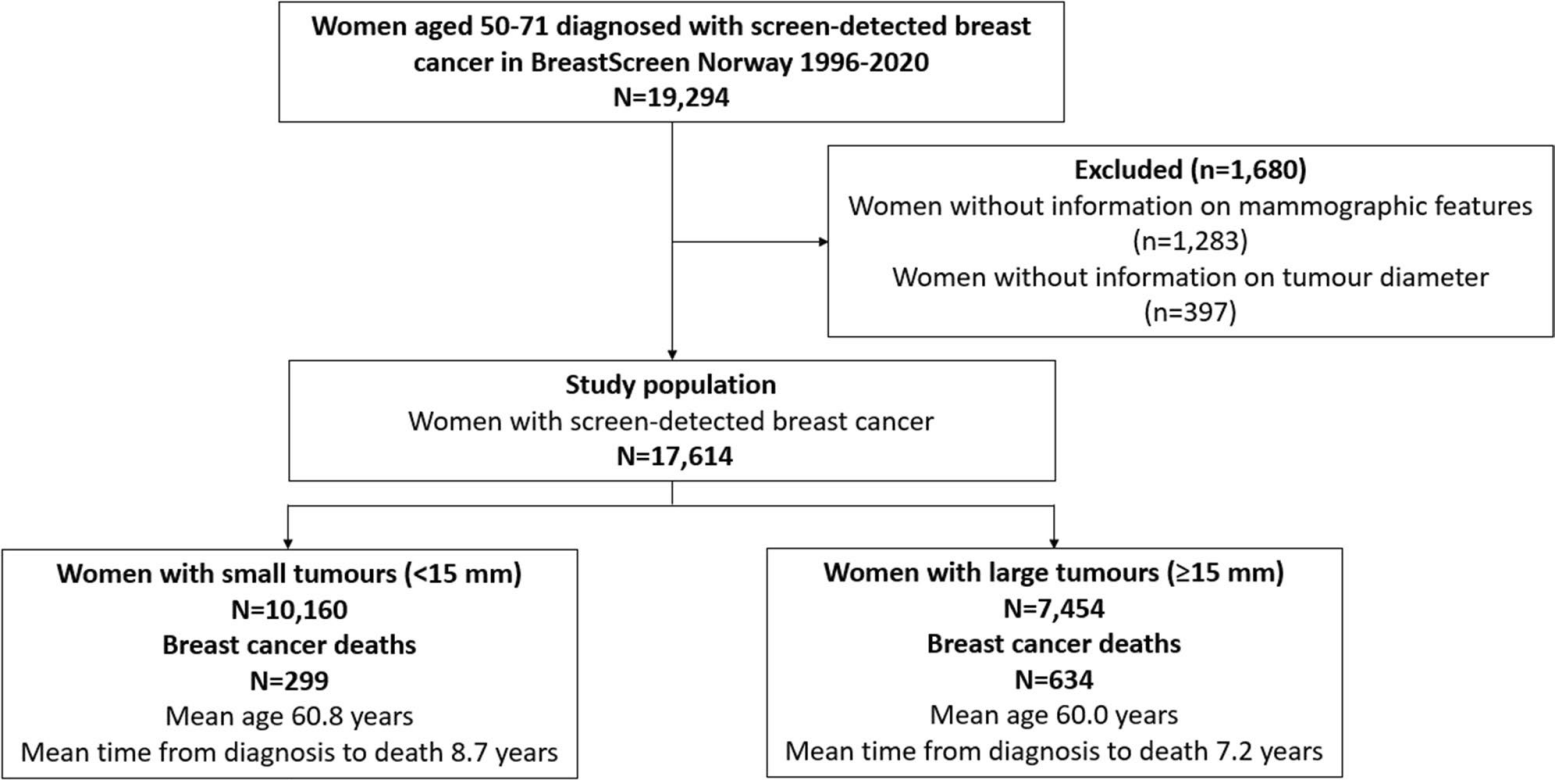
Study sample with exclusions

We included screen-detected cancer, defined as primary invasive breast cancer (breast carcinoma of no special type, invasive lobular carcinoma and other invasive breast tumours) diagnosed after a positive screening examination (Fig. 1). Mammographic features included mass, spiculated mass, architectural distortion, asymmetric density, density with calcification and calcification alone [3, 4]. Information about mammographic features was extracted from the radiology forms reported for all recalled cases in BreastScreen Norway.

Mass was defined as a space-occupying lesion, visible in two different projections, characterised by its contour, including circumscribed, microlobulated, masked, or indistinct [3, 4, 17, 23]. Spiculated mass was defined as an opacity formed by a dense centre from which multiple linear radial prolongations called spicules arise, i.e. a lesion with spiculated or stellate margins [4]. Asymmetric density was defined as areas of increased focal density without the discrete borders of a mass [3]. Density with calcification was defined as an area of increased focal density with accompanying calcifications [4]. Calcification alone was defined as calcifications without any associated increased density [4]. The term architectural distortion was included in the radiology forms since 2010 and was defined as a distortion of normal breast architecture without the identification of a discrete mass [4, 24].

The Norwegian Cause of Death Registry regularly provides the Cancer Registry with information about date and cause of death. The last update included deaths prior to January 1, 2022 [25]. Women were followed up from date of their breast cancer diagnosis between the 1st of January 1996 and the 31st of December 2020, until death from breast cancer or until the 31st of December 2020. Women, who died of other causes than breast cancer or emigrated during 1996–2020, contributed with life-years to the study and were censored at death or emigration.

Information about age, mammographic density, tumour diameter (mm), histologic grade (1–3 by Nottingham scale) [26], lymph node status (positive or negative), estrogen, progesterone and human epidermal growth factor receptor 2 (HER2) status [27] was extracted from the Cancer Registry of Norway database. Data on HER2 status and immunohistochemical subtypes (luminal A, luminal B HER2−, luminal B HER2+, HER2+ and triple negative) was available for the period 2010–2020. Data on tumour diameter was based on the pathologist’s reporting of the largest tumour measure and categorised as small (<15 mm) and large (≥15 mm) based on previous studies [6, 9, 28]. In 1996–2012, mammographic density of women recalled for further assessment was categorised by the percentage of fibroglandular tissue on the mammogram: (1) <30%, (2) 30–70%, (3) >70% [29]. The classification was replaced by BI-RADS 5th edition [3] in 2013. For this study, data on the three-category classification was used from women screened in 1996–2012, while data from the BI-RADS density classification, used 2013–2020, was recategorised to match the three-category classification; BI-RADS a was categorised as mammographic density 1; b and c as density 2; and d as density 3. The validity of this re-categorisation is described elsewhere [30].

### Statistical analysis

Distribution of mammographic features for small versus large tumours was presented as numbers and percentages. Cox regression was used to estimate hazard ratios (HR) with 95% confidence intervals (CI) for breast cancer death associated with mammographic features for small and large tumours, separately, and for the entire study population. Spiculated mass was used as the reference category as this feature was the most numerous. We adjusted for age at diagnosis, tumour diameter and lymph node status, dichotomised in negative and positive. Cumulative hazards for breast cancer death were presented in Nelson-Aalen plots, stratified by mammographic features for small and large tumours. Follow-up time was 25 years for tumours with all features except architectural distortion which has been registered since 2010, with a combined follow-up of 8 years for small tumours and 11 years for large tumours. Numbers and percentages of women for mammographic density, histologic grade and lymph node, estrogen and progesterone receptor status for women with small versus large tumours, as well as mean age with standard deviation (SD) and median tumour diameter with interquartile range (IQR) were presented by mammographic features for small and large tumours separately. Further, the same descriptive information, as well as numbers and percentages of women for HER2 status and immunohistochemical subtypes (available for women diagnosed 2010–2020), was presented for the entire study population in a supplemental table. Descriptive information on HER2 status and immunohistochemical subtypes and mean time from breast cancer diagnosis to death with SD were shown by mammographic features for small versus large tumours in supplemental tables. Descriptive information on age (mean, SD) and histopathologic tumour characteristics was also presented for women who died from breast cancer stratified by mammographic features for small versus large tumours in a supplemental table. All analyses were performed with Stata [31].

## Results

Data from 17,614 women diagnosed with invasive screen-detected breast cancer and 933 breast cancer deaths was included in the analyses (Fig. 1): 10,160 women and 299 deaths among women with small tumours, and 7454 women and 634 deaths among those with large tumours. Mean age was 60.8 (SD=5.8) and 60.0 (SD=5.8) years for women with small and large tumours, respectively. Mean time from diagnosis to death was 8.7 (SD=5.0) years for women with small tumours and 7.2 (SD=4.6) years for women with large tumours. The proportions of mass and of calcification alone were higher for women with small versus large tumours (26.7 versus 17.8% for mass and 12.3 versus 6.3% for calcification alone, respectively) (Table 1).

**Table 1:** Distribution of mammographic features for women with invasive screen-detected breast cancer diagnosed 1996–2020, stratified by tumour diameter; small tumours (< 15 mm) and large tumours (≥ 15 mm)

Mammographic features	Small tumours (< 15 mm)	Large tumours (≥ 15 mm)	Total	*p*-value*
	(*n*=10,160)	(*n*=7454)	(*n*=17,614)	
	*N* (%)	*N* (%)	*N* (%)	<0.001
Mass	2716 (26.7)	1330 (17.8)	4046 (23.0)	<0.001
Spiculated mass	3741 (36.8)	3177 (42.6)	6918 (39.3)	<0.001
Architectural distortion	242 (2.4)	181 (2.4)	423 (2.4)	0.843
Asymmetric density	1438 (14.2)	1407 (18.9)	2845 (16.1)	<0.001
Density with calcification	774 (7.6)	886 (11.9)	1660 (9.4)	<0.001
Calcification alone	1249 (12.3)	473 (6.3)	1722 (9.8)	<0.001

^*^p value is based on a chi-square test comparing women with small and large tumours

Using spiculated mass as reference, the highest adjusted HR for breast cancer death was 2.48 (95% CI 1.67–3.68) for calcification alone among women with small tumours (Table 2). The highest adjusted HR for large tumours was shown for density with calcification, 1.30 (95% CI 1.02–1.66).

**Table 2:** Hazard ratios (HR) with 95% confidence intervals (CI) of Cox regression showing the risk of breast cancer death by mammographic features for women diagnosed with invasive screen-detected cancer in BreastScreen Norway, 1996–2020, stratified by tumour diameter

	Small tumours (< 15 mm)	Large tumours (≥ 15 mm)	All women
	Unadjusted HR (95% CI) (*n*= 10,160)	Unadjusted HR (95% CI) (*n*=7454)	Unadjusted HR (95% CI) (*n*=17,614)
Mammographic feature			
Spiculated mass	1.00	1.00	1.00
Mass	1.09 (0.81, 1.48)	1.18 (0.85, 1.56)	1.00 (0.84, 1.20)
Architectural distortion	0.00 (-)	1.23 (0.54, 2.78)	0.97 (0.43, 2.18)
Asymmetric density	1.57 (1.12, 2.22)	1.37 (1.11, 1.68)	1.53 (1.28, 1.83)
Density with calcification	1.49 (0.98, 2.25)	1.39 (1.09, 1.78)	1.53 (1.24, 1.89)
Calcification alone	1.64 (1.14, 2.34)	1.02 (0.70, 1.49)	1.04 (0.81, 1.34)
Age	1.01 (0.99, 1.03)	1.02 (1.00, 1.03)	1.01 (0.99, 1.02)
Tumour diameter	1.10 (1.06, 1.14)	1.03 (1.02, 1.03)	1.04 (1.03, 1.04)
Positive lymph node status	2.77 (2.15, 3.58)	2.68 (2.28, 3.14)	3.52 (3.10, 4.01)
	Adjusted* HR (95% CI) (*n*=9915)	Adjusted* HR (95% CI) (*n*=7389)	Adjusted* HR (95% CI) (*n*=17,304)
Mammographic feature			
Spiculated mass	1.00	1.00	1.00
Mass	1.20 (0.89, 1.63)	1.25 (1.00, 1.54)	1.15 (0.97, 1.38)
Architectural distortion	0.00 (–)	1.09 (0.47, 2.46)	0.85 (0.38, 1.91)
Asymmetric density	1.65 (1.16, 2.34)	1.22 (0.99, 1.51)	1.31 (1.09, 1.57)
Density with calcification	1.58 (1.04, 2.41)	1.30 (1.02, 1.66)	1.35 (1.09, 1.68)
Calcification alone	2.48 (1.67, 3.68)	1.06 (0.73, 1.56)	1.29 (0.99, 1.67)
Age	1.02 (1.00, 1.05)	1.02 (1.01, 1.04)	1.02 (1.01, 1.03)
Tumour diameter	1.10 (1.06, 1.15)	1.02 (1.02, 1.03)	1.03 (1.02, 1.03)
Positive lymph node status	2.48 (1.91, 3.21)	2.51 (2.13, 2.95)	2.82 (2.46, 3.24)

^*^Adjusted for age, tumour diameter, and lymph node status

**Fig. 2:**
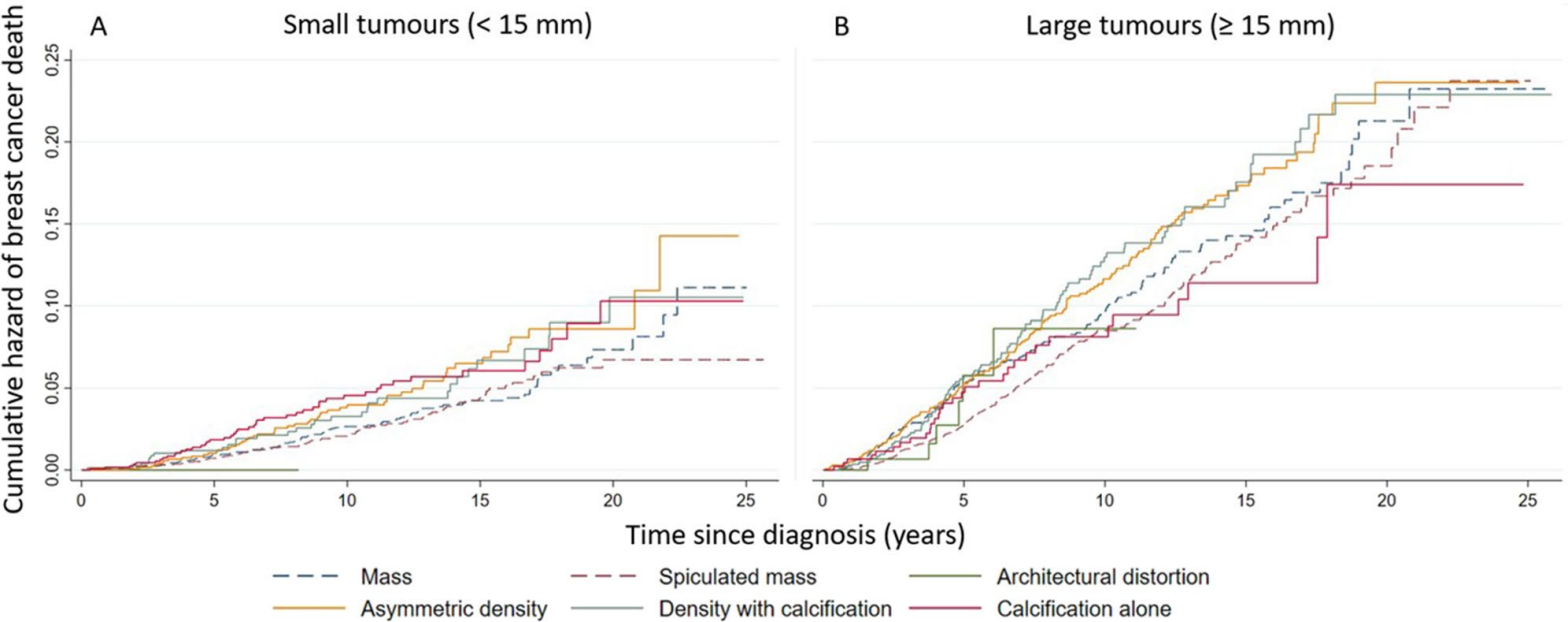
Nelson-Aalen plots showing the cumulative hazard of breast cancer death for various mammographic features over 25 years among women with invasive screen-detected tumours diagnosed 1996–2020, stratified by tumour diameter. **A** Left panel: For small tumours (< 15 mm); **B** Right panel: For large tumours (≥ 15 mm)

For women with small tumours, the cumulative risk of dying from breast cancer after 25 years of follow-up was about 15% for women with asymmetric density and 10–12% for women with calcification alone (Fig. 2A). The cumulative risk of dying from breast cancer was higher for women with large tumours compared to women with small tumours and was 23–24% for women with density with calcification, mass, spiculated mass and asymmetric density (Fig. 2B).

For women with small tumours, 11.3% of the cases classified as calcification alone were assigned mammographic density category 3 compared to 3.2% of masses and 4.6% of spiculated masses (*p*<0.001 for both) (Table 3). Histologic grade 3 tumours were present in 39.8% of calcification alone, while the percentage was 15.2% for all mammographic features of small tumours (*p*<0.001). The proportion of Luminal A subtype was lower for calcification alone (49.3%) compared to mass (66.5%), spiculated mass (65.9%), architectural distortion (69.5%) and asymmetric density (65.0%) (*p*<0.001 for all) (Table A3A).

**Table 3:** Descriptive information on age and histopathologic tumour characteristics for women with invasive screen-detected breast cancer diagnosed 1996–2020, stratified by mammographic features, for small tumours (< 15 mm)

	Mass *n*=2716 (26.7%)	Spiculated mass *n*=3741 (36.8%)	Architectural distortion *n*=242 (2.4%)	Asymmetric density *n*=1438 (14.2%)	Density with calcification *n*=774 (7.6%)	Calcification alone *n*=1249 (12.3%)	Total *n*=10,160
Age, mean (SD) years	61.5 (5.5)	60.8 (5.8)	61.4 (5.9)	60.9 (5.7)	59.8 (5.9)	59.2 (6.0)*^&^	60.8 (5.8)
Mammographic density, *n* (%)							
1	902 (35.8)	923 (26.0)	38 (16.1)	289 (21.1)	168 (23.0)	170 (14.5)^#¤§^^	2490 (26.0)
2	1537 (61.0)	2464 (69.4)	182 (77.1)	968 (70.8)	504 (69.0)	873 (74.2)^#^	6528 (68.1)
3	82 (3.2)	163 (4.6)	16 (6.8)	111 (8.1)	59 (8.1)	133 (11.3)^#¤^	564 (5.9)
Data not available, *n*	195	191	6	70	43	73	578
Tumour diameter, median (IQR) mm	9.0 (0.5–14.0)	10.0 (0.9–14.0)	10.0 (2.5–14.0)	10.0 (1.0–14.0)	10.0 (0.9–14.0)	5.0 (0.1–14.0)	9.0 (0.1–14.0)
Histologic grade, *n* (%)							
1	1091 (40.5)	1776 (47.7)	115 (47.5)	567 (40.2)	274 (36.0)	265 (22.5)^#^	4088 (40.8)**
2	1193 (44.3)	1649 (44.3)	108 (44.6)	658 (46.7)	342 (44.9)	446 (37.8)	4396 (43.9)
3	407 (15.1)	301 (8.1)	19 (7.9)	185 (13.1)	145 (19.1)	469 (39.8)^#¤&§^^	1526 (15.2)**
Data not available, *n*	25	15	0	28	13	69	150
Lymph node status, *n* (%)							
Positive	261 (9.9)	463 (12.5)	20 (8.3)	194 (13.8)	98 (13.0)	129 (11.0)	1165 (11.8)
Data not available, *n*	66	52	2	27	21	76	244
Hormonal status, *n* (%)							
ER positive	2347 (89.1)	3492 (95.5)	229 (95.0)	1277 (91.6)	647 (88.6)	937 (84.0)^¤&^	8929 (91.4)
Data not available, *n*	81	84	1	44	44	133	387
PR positive	1905 (72.9)	2791 (77.0)	197 (82.4)	1031 (74.2)	494 (68.0)	646 (58.7)^#¤&§^^	7064 (72.9)
Data not available, *n*	101	117	3	49	48	148	466

*SD* standard deviation, *IQR* interquartile range, *ER* estrogen receptor, *PR* progesterone receptor

^*^p<0.001 for *t*-test comparison of means for mass versus calcification alone

^#^*p*<0.001 for chi-square test for comparison of mass versus calcification alone

^¤^*p*<0.001 for chi-square test for comparison of spiculated mass versus calcification alone

^&^*p*<0.001 for chi-square test for comparison of architectural distortion versus calcification alone

^§^*p*<0.001 for chi-square test for comparison of asymmetric density versus calcification alone

^^^*p*<0.001 for chi-square test for comparison of density with calcification versus calcification alone

^**^p<0.001 for chi-square test for comparison of calcification alone versus total

For women with large tumours, histologic grade 3 tumours were found in 48.3% of the cases classified as calcification alone while it was 26.0% for all mammographic features (*p*<0.001) (Table 4). The proportion of lymph node positive tumours was higher for density with calcification (41.4%) compared to calcification alone (30.1%) (*p*<0.001).

**Table 4:** Descriptive information on age and histopathologic tumour characteristics for women with invasive screen-detected breast cancer diagnosed 1996–2020, stratified by mammographic features, for large tumours (≥ 15 mm)

	Mass *n*=1330 (17.8%)	Spiculated mass *n*=3177 (42.6%)	Architectural distortion *n*=181 (2.4%)	Asymmetric density *n*=1407 (18.9%)	Density with calcification *n*=886 (11.9%)	Calcification alone *n*=473 (6.3%)	Total *n*=7454
Age, mean (SD) years	60.4 (5.7)	60.0 (5.9)	60.5 (6.5)	60.3 (5.7)	59.3 (5.8)	58.9 (5.9)*	60.0 (5.8)
Mammographic density, *n* (%)							
1	399 (32.5)^~^	599 (19.8) ^$^	11 (6.4)	241 (18.5)^£^	142 (17.3)^@^	40 (9.1)^#^	1432 (20.5)
2	763 (62.1)^~^	2228 (73.6)^$^	153 (89.0)	912 (69.8)^£^	590 (71.8)^@^	346 (78.6)^#^	4992 (71.4)
3	67 (5.5)	199 (6.6)	8 (4.7)	153 (11.7)	90 (11.0)	54 (12.3)^#&^	571 (8.2)
Data not available, *n*	101	151	9	101	64	33	459
Tumour diameter, median (IQR) mm	19.0 (15.0–80.0)	20.0 (15.0–90.0)	21.0 (15.0–80.0)	20.0 (15.0–90.0)	21.0 (15.0–98.0)	20.0 (15.0–90.0)	20.0 (14.7–98.0)
Histologic grade, *n* (%)							
1	212 (16.1)	671 (21.3)	42 (23.5)	270 (19.4)	145 (16.5)	45 (9.6)^¤&§^	1385 (18.8)
2	626 (47.6)	1892 (60.1)	107 (59.8)	815 (58.7)	436 (49.6)	197 (42.1)^¤&§^	4073 (55.2)
3	476 (36.2)	587 (18.6)	30 (16.8)	304 (21.9)	299 (34.0)	226 (48.3)^#¤&§^^	1922 (26.0)**
Data not available, *n*	16	27	2	18	6	5	74
Lymph node status, *n* (%)							
Negative	907 (68.9)	2085 (66.4)	122 (67.4)	852 (60.8)	516 (58.6)	320 (69.9)	4812 (65.1)
Positive	410 (31.1)	1058 (33.6)	59 (32.6)	549 (39.2)	364 (41.4)	138 (30.1)^^^	2578 (34.9)
Data not available, *n*	13	24	0	6	6	15	64
Hormonal status, *n* (%)							
ER positive	1033 (79.7)^~%^	2931 (93.6)	170 (94.4)	1220 (88.9)	752 (86.4)^@+^	352 (80.4)^&¤^	6458 (88.6)
Data not available, *n*	34	46	1	34	18	35	168
PR positive	821 (63.6)	2403 (77.2)	143 (79.4)	949 (69.5)	578 (66.8)^@+^	260 (59.8)^¤&§^	5154 (71.1)
Data not available, *n*	39	66	1	41	21	38	206

*SD* standard deviation, *IQR* interquartile range, *ER* estrogen receptor, *PR* progesterone receptor

^*^p<0.001 for *t*-test comparison of means for mass versus calcification alone

^~^*p*<0.001 for chi-square test for comparison of mass versus architectural distortion

^$^*p*<0.001 for chi-square test for comparison of spiculated mass versus architectural distortion

^£^*p*<0.001 for chi-square test for comparison of architectural distortion versus asymmetric density

^@^*p*<0.001 for chi-square test for comparison of architectural distortion versus density with calcification

^#^*p*<0.001 for chi-square test for comparison of mass versus calcification alone

^&^*p*<0.001 for chi-square test for comparison of architectural distortion versus calcification alone

^¤^*p*<0.001 for chi-square test for comparison of spiculated mass versus calcification alone

^§^*p*<0.001 for chi-square test for comparison of asymmetric density versus calcification alone

^**^p<0.001 for chi-square test for comparison of calcification alone versus total

^^^*p*<0.001 for chi-square test for comparison of density with calcification versus calcification alone

^%^*p*<0.001 for chi-square test for comparison of spiculated mass versus mass

^+^*p*<0.001 for chi-square test for comparison of spiculated mass versus density with calcification

For 299 women with small tumours who died, 28.8% had mass, 27.1% spiculated mass, 18.1% asymmetric density, 10.4% density with calcification and 15.7% calcification alone (Table A4A). Histologic grade 3 tumours were present in 40.0% of calcification alone, while it was 23.7% for all mammographic features (*p*=0.02). For 634 women with large tumours who died, 31.9% had spiculated mass, 22.6% mass, 24.9% asymmetric density, 7.6% density with calcification and 4.9% calcification alone (Table A4B). Histologic grade 3 tumours were present in 64.5% of calcification alone, while it was 37.7% for all mammographic features (*p*=0.003).

## Discussion

In our study population of 17,614 women with invasive screen-detected breast cancer, we found small tumours presenting as calcification alone, and large tumours presenting as density with calcification to have the highest risk of breast cancer death in a Cox regression model with 25 years of follow up. Our hypothesis was thus confirmed.

Women with small tumours had higher proportions of mass and calcification alone and lower proportions of spiculated mass, asymmetric density and density with calcification compared to women with large tumours. This might suggest that the distribution of mammographic features changed with increasing tumour diameter and progression [32]. Histologic grade 3 was more common for calcification alone compared to the other features for women with small tumours and for those who died, and might be of influence for the severe outcome [26]. Further, tumours presenting as calcification alone or in combination with density or asymmetric density might have been missed or dismissed when the tumour was smaller (< 5 mm) [33, 34]. Tumours only identifiable because of calcification alone could be missed at prior screening if the suspicious calcification component was not formed yet [11, 35, 36]. High mammographic density might have obscured tumours presenting as density with calcification or asymmetric density in the prior screening round [18, 19, 34, 37, 38]. Calcifications in association with mass or asymmetric density were reported to be more common on prior mammograms of missed screen-detected cancers than other mammographic features in a review study [34]. Calcification without associated mass or density on a screening mammogram is usually associated with ductal carcinoma in situ (DCIS), which results in a different treatment pathway compared to invasive breast cancer [39–41]. However, fragmented or dotted casting calcifications types might be associated with an invasive component [42], and we assume that these cases were included in the groups of calcification alone and density with calcification. Furthermore, studies have reported casting calcifications to be common in high-grade DCIS, while small clusters of punctate or granular calcifications to be more common in low-grade DCIS, and both types could be associated with an invasive component [6, 28, 35]. Unfortunately, we were not able to distinguish between the different calcification types. However, the presence of high-grade DCIS in addition to a small invasive component might lead to more aggressive and extensive disease and therefore less favourable prognosis, specifically if the tumour was missed at mammography or not identified in the tissue sample by pathologists [6, 28, 35]. As the malignant process is highly associated with the usually more extended intraductal processes (DCIS), causing casting calcifications in parts of the affected tissue, the size of calcification areas on a mammogram is a more precise but potentially even too small estimate of the actual extent of the intraductal processes. The size of the histologically proven invasive tumour by that probably systematically underestimates the size and number of potentially invasive components that might be found in other areas of the mammographic distribution of calcification [6, 35, 42].

Women with large tumours presenting as density with calcification had a higher risk of breast cancer death and the highest proportion of lymph node positive tumours compared to those with tumours presenting as spiculated mass. However, the highest proportions of histologic grade 3 tumours and high mammographic density were observed for calcification alone. Tumours with a large diameter presenting as density with calcification might be missed at prior screening due to less visible invasive components and/or no formed calcifications [33, 34, 36].

Our findings indicate that calcification alone in small tumours, which might represent an extensive and possibly underestimated size of high-grade DCIS in addition to an invasive component, could result in a less favourable prognosis. However, to consider changing in the treatment algorithm, including extensive surgical treatment in addition to radiotherapy for women with calcification alone, more knowledge is needed on the histopathologic characteristics and molecular biomarkers associated with tumour growth and mammographic features [43, 44].

Small (<15 mm and <10 mm) stellate and spiculated screen-detected tumours are associated with favourable survival [6, 9, 10]. Results from our study support these findings. The association between high risk of breast cancer death and casting or pleomorphic calcifications alone is shown for tumours of <15 mm [6, 11, 35, 42]. Results from our study are in line with these findings; however, we did not include calcification types in the analyses. A study from Sweden has shown that architectural distortion was associated with triple negative breast cancer and thereby a higher risk of breast cancer death [8]. Our results do not support these findings (Supplemental table A3). This might be due to a small number of cases with architectural distortion (2.4%), as its reporting started in 2010, or due to a high number of missing values for immunohistochemical subtypes registered from 2010 and on. Furthermore, invasive tumours presenting as architectural distortion are commonly large in diameter and symptomatic, and architectural distortion associated with small screen-detected tumours is rare [8]. According to a Swedish study, the distribution of mammographic features had changed during the period from 1996 to 2010; an increasing rate of tumours <15 mm as well as malignant calcifications and architectural distortion on the cost of spiculated and circumscribed masses was shown [32]. Our results might have also been influenced by these changes.

Solely women with data on tumour diameter at pathology reports following primary surgical treatment were included. Women receiving neoadjuvant therapy were excluded as their tumour diameter was not estimated by pathologists at diagnosis. Multifocal and multicentric tumours were not investigated as we used information solely from the women with one invasive tumour. However, it is possible that multifocal and/or multicentric tumours were missed in women with dense breasts, as use of supplementary MRI or contrast-enhanced mammography was not a standard procedure in the beginning of the study period. However, in 2021, supplementary MRI was performed for diagnosing 29.7% of all breast cancer cases in Norway [45]. We used registry data, and the radiologists’ subjective interpretation and reporting of mammographic features was not reviewed or validated, as the review is costly and time-consuming. The subjectivity in the reporting could have resulted in classification of masses and architectural distortions as asymmetric density and vice versa, which could be of influence for the distribution of features [46]. Further, a misclassification of tumours having density with calcification as calcification alone in a dense breast might have occurred despite the clear definition of density with calcification as an area of increased focal density with accompanying calcifications which could not be interpreted as an entirely dense breast with calcifications alone or parts of dense tissue unassociated with calcifications. The use of screen-film mammography from 1996 to 2000–2011, transition from screen-film to digital mammography in 2000–2011 and further use of digital mammography since 2011 might have affected the subjective interpretation of mammographic features [47]. Furthermore, the distribution of mammographic features over time might have been influenced by improvements in diagnostics, histologic types and immunohistochemical subtypes of breast cancer, leading to higher proportions of calcifications and asymmetries compared to masses; however, we were unable to track these changes in the study [13, 32, 36]. Architectural distortion, asymmetric density and calcification alone might have been better visible and more often reported in digital versus screen-film mammography [48]. Information on types of calcifications was not available, and the effect of casting calcifications on the risk of breast cancer death might have been underestimated, while the effect of other calcifications types might have been overestimated [49]. The study population had about 20–26% of women with density category 1, which might be associated with the age of the women [30]. Although the subjective mammographic density classification was different from BI-RADS in our study, the category 3 was assumed to be similar to BI-RADS d, as reported in a previous study comparing the Norwegian three-category and BI-RADS four-category classification [30]. Therefore, density category 3 might have been linked to a more aggressive disease and worse prognosis; however, studies did not confirm the direct association with prognosis [19, 50, 51]. High mammographic density was more likely associated with missed tumours or tumours detected in a late stage, which might be of influence for the prognosis [34, 37, 52]. Lack of complete information on tumours’ histopathology and biomarkers for the study period hampers the definitve conclusions on calcificiation alone or density with calcification as an independent factor for risk of breast cancer death. The highest cumulative risk of dying from breast cancer was shown for women with small tumours presented as asymmetric density. However, the cumulative risk was not considered the main outcome as the risk was unadjusted and associated with high uncertainty at the end of the follow up.

Invasive screen-detected tumours of <15 mm presenting as calcification alone and tumours of ≥15 mm presenting as density with calcification were associated with a higher risk of breast cancer death compared to those presenting as spiculated mass among women attended BreastScreen Norway, 1996–2020.

### Supplementary Information

Below is the link to the electronic supplementary material.

Supplementary file1 (PDF 313 KB)

## Data Availability

Data used in the analyses can be made available on request to https://helsedata.no/, given legal basis in Articles 6 and 9 of the GDPR and that the processing is in accordance with Article 5 of the GDPR.

## Abbreviations

BI-RADS: Breast Imaging-Reporting and Data System
CI: Confidence interval
DCIS: Ductal carcinoma in situ
HER2: Human epidermal growth factor receptor 2
HR: Hazard ratio
IQR: Interquartile range
MRI: Magnetic resonance imaging
SD: Standard deviation

## Declarations

### Disclaimer

Data from the Cancer Registry of Norway (CRN) has been used in this publication. The interpretation and reporting of these data are the sole responsibility of the authors, and no endorsement by CRN is intended nor should be inferred.

### Guarantor

The scientific guarantor of this publication is Solveig Hofvind.

### Statistics and biometry

The statistical analyses were planned and performed by Elin W. Bjornson and critically reviewed by Marthe Larsen, both have significant statistical expertise.

### Study subjects or cohorts overlap

Women with tumours < 10 mm (*n*=5152) were reported in a Research Presentation at the European Congress of Radiology 05.05.2023.

Study subjects or cohorts have not been reported elsewhere.

### Methodology

• retrospective

• performed at one institution
