# Supplementary material for: Mammographic features and risk of breast cancer death among women with invasive screen-detected cancer in BreastScreen Norway 1996–2020
Source: Eur Radiol. 2023 Nov 8;34(5):3364–74. doi: 10.1007/s00330-023-10369-w (PMC11126444; doi:10.1007/s00330-023-10369-w)
Supplement: Supplementary file 1 — Supplementary file1 (PDF 313 KB) [file 330_2023_10369_MOESM1_ESM.pdf]

Mammographic features and risk of breast cancer death among women with invasive screen-detected cancer in BreastScreen Norway 1996-2020

Electronic Supplementary Material

Table A1. Descriptive information on age and tumour characteristics for women with invasive screen-detected breast cancer diagnosed 1996-2020

	Total
	17,614 (100.0%)
Age, mean (SD) years	60.4 (5.8)
Mammographic density, n (%)	
1	3922 (23.7)
2	11520 (69.5)
3	1135 (6.9)
Data not available, n	1037
Tumour diameter, median (IQR) mm	13.0 (0.1-130.0)
Histologic grade, n (%)	
1	5473 (31.5)
2	8469 (48.7)
3	3448 (19.8)
Data not available, n	224
Lymph node status, n (%)	
Positive	3743 (21.6)
Data not available, n	308
Estrogen receptor status, n (%)	
Positive	15387 (90.2)
Data not available, n	555
Progesterone receptor status, n (%)	
Positive	12218 (72.1)
Data not available, n	672
HER2 status, n (%)	
Positive	2066 (19.5)
Data not available, n	7021
Subtype, n (%)	
Luminal A	6455 (61.5)
Luminal B HER2-	1437 (13.7)
Luminal B HER2+	1734 (16.5)
HER2+	318 (3.0)
Triple negative	549 (5.2)
Data not available, n	7121

SD – standard deviation; IQR – interquartile range; ER – estrogen receptor; PR – progesterone receptor, HER2 - human epidermal growth factor receptor 2

Table A2. Mean time from breast cancer diagnosis to death for women with small (< 15mm) versus large tumours (≥ 15 mm), and all women died following a diagnosis of invasive screen-detected breast cancer 1996-2020

	Years from breast cancer diagnosis to death (mean, standard deviation)		
Mammographic feature	Small tumours (< 15 mm) n=299 (32.0 %)	Large tumours (≥ 15 mm) n=634 (68.0 %)	Total n=933 (100.0 %)
Mass	9.3 (5.3)	7.1 (4.8)	7.9 (5.1)
Spiculated mass	8.9 (4.8)	7.8 (4.7)	8.1 (4.8)
Architectural distortion	0	4.2 (1.5)	4.2 (1.5)
Asymmetric density	8.8 (4.8)	6.9 (4.6)	7.4 (4.7)
Density with calcification	8.9 (5.5)	6.9 (4.3)	7.4 (4.7)
Calcification alone	7.4 (4.8)	6.0 (4.5)	6.9 (4.7)
Total	8.7 (5.0)	7.2 (4.6)	7.7 (4.8)

Table A3. Descriptive information on HER2 and breast cancer subtypes for women with invasive screen-detected breast cancer diagnosed 2010-2020, stratified by mammographic features. **A.** For small tumours (< 15 mm); **B.** For large tumours (≥ 15 mm).

**A**	**Mass** n=2716 (26.7 %)	**Spiculated mass** n=3741 (36.8 %)	**Architectural distortion** n=242 (2.4%)	**Asymmetric density** n=1438 (14.2 %)	**Density with calcification** n=774 (7.6%)	**Calcification alone** n=1249 (12.3 %)	**Total** n=10160 (100.0 %)
HER2 status, n (%)							
Positive	185 (14.0)	416 (16.9)	46 (19.1)	153 (17.3)	96 (23.8)	190 (26.1) [#¤`§]	1086 (17.9)
Data not available, n	1388	1276	1	555	370	521	4111
Subtype, n (%)							
Luminal A	874 (66.5)	1616 (65.9)	166 (69.5)	566 (65.0)	228 (56.7)	353 (49.3) [#¤&§]	3803 (63.5)
Luminal B HER2-	158 (12.0)	353 (14.4)	20 (8.4)	118 (13.6)	56 (13.9)	135 (18.9) [&]	840 (14.0)
Luminal B HER2+	150 (11.4)	379 (15.5)	41 (17.2)	133 (15.3)	75 (18.7)	133 (18.6)	911 (15.2)
HER2+	33 (2.5)	35 (1.4)	5 (2.1)	19 (2.2)	21 (5.2)	54 (7.5) [#¤&§]	167 (2.8)
Triple negative	99 (7.5)	68 (2.8)	7 (2.9)	35 (4.0)	22 (5.5)	41 (5.7)	272 (4.5)
Data not available, n	1402	1290	3	567	372	533	4167

HER2 - human epidermal growth factor receptor 2
[#] p<0.001 for chi-square test for comparison of mass versus calcification alone
[¤] p<0.001 for chi-square test for comparison of spiculated mass versus calcification alone
[&] p<0.001 for chi-square test for comparison of architectural distortion versus calcification alone
[§] p<0.001 for chi-square test for comparison of asymmetric density versus calcification alone
[`] p= 0.02 for chi-square test for comparison of architectural distortion versus calcification alone

**B**	**Mass** n=1330 (17.8 %)	**Spiculated mass** n=3177 (42.6 %)	**Architectural distortion** n=181 (2.4 %)	**Asymmetric density** n=1407 (18.9 %)	**Density with calcification** n=886 (11.9 %)	**Calcification alone** n=473 (6.3 %)	**Total** n=7454 (100.0 %)
HER2 status, n (%)							
Positive	107 (18.3)	431 (19.6)	38 (21.1)	162 (21.6)	148 (28.7)	94 (30.2) [#¤]	980 (21.6)
Data not available, n	756	994	2	664	373	165	2954
Subtype, n (%)							
Luminal A	307 (53.5)	1375 (63.0)	114 (63.7)	441 (59.3)	272 (53.0)	143 (46.4) [¤&§]	2652 (58.9)
Luminal B HER2-	77 (13.4)	286 (13.1)	19 (10.6)	104 (14.0)	67 (13.1)	44 (14.3)	597 (13.3)
Luminal B HER2+	76 (13.2)	390 (17.9)	37 (20.7)	136 (18.3)	119 (23.2)	65 (21.1)	823 (18.3)
HER2+	30 (5.2)	39 (1.8)	1 (0.6)	26 (3.5)	28 (5.5)	27 (8.8) [¤&§]	151 (3.4)
Triple negative	84 (14.6) [%~]	93 (4.3)	8 (4.5)	36 (4.9)	27 (5.3)	29 (9.4)	277 (6.2)
Data not available, n	756	3177	181	1407	886	473	411

HER2 - human epidermal growth factor receptor 2
[#] p<0.001 for chi-square test for comparison of mass versus calcification alone
[¤] p<0.001 for chi-square test for comparison of spiculated mass versus calcification alone
[&] p<0.001 for chi-square test for comparison of architectural distortion versus calcification alone
[§] p<0.001 for chi-square test for comparison of asymmetric density versus calcification alone
[^] p<0.001 for chi-square test for comparison of density with calcification versus calcification alone
[~] p<0.001 for chi-square test for comparison of mass versus architectural distortion
[%] p<0.001 for chi-square test for comparison of spiculated mass versus mass

Table A4. Descriptive information on age and tumour characteristics for women who died following a diagnosis of invasive screen-detected breast cancer 1996-2020, stratified by mammographic features. **A.** For small tumours (< 15 mm); **B.** For large tumours (≥ 15 mm); and **C.** for all

**A.** Small tumours (<15 mm)	**Mass** n=86 (28.8 %)	**Spiculated mass** n=81 (27.1 %)	**Architectural distortion** n=0 (0 %)	**Asymmetric density** n=54 (18.1 %)	**Density with calcification** n=31 (10.4 %)	**Calcification alone** n=47 (15.7 %)	**Total** n=299 (100.0 %)
Age, mean (SD) years	62.1 (5.6)	60.1 (6.1)		60.3 (5.5)	60.9 (5.0)	58.7 (6.3)	60.6 (5.8)
Mammographic density, n (%)							
1	26 (35.1)	33 (45.8)		13 (25.5)	8 (27.6)	6 (14.6) [&§]	86 (32.2)
2	41 (55.4)	35 (48.6)		34 (66.7)	19 (65.5)	27 (65.9)	156 (58.4)
3	7 (9.5)	4 (5.6)		4 (7.8)	2 (6.9)	8 (19.5) [§]	25 (9.4)
Data not available, n	12	9		3	2	6	32
Tumour diameter, median (IQR) mm	11.0 (5.0-14.0)	11.0 (6.0-14.0)		10.0 (4.0-14.0)	11.0 (7.0-13.0)	8.0 (1.0-13.0)	11.0 (1.0-14.0)
Histologic grade, n (%)							
1	25 (29.1)	27 (33.3)		16 (30.2)	7 (22.6)	5 (11.1)	80 (27.0)**
2	40 (46.5)	45 (55.6)		23 (43.4)	16 (51.6)	22 (48.9)	146 (49.3)
3	21 (24.4)	9 (11.1)		14 (26.4)	8 (25.8)	18 (40.0) [§]	70 (23.7)**
Data not available	0	0		1	0	2	3
Lymph node status, n (%)							
Positive	22 (26.5)	28 (35.9)		15 (28.3)	4 (13.3)	14 (32.6)	83 (28.9)
Data not available, n	3	3		1	1	4	12
Hormonal status, n (%)							
ER positive	70 (86.4)	64 (87.7)		42 (84.0)	18 (64.3)	31 (79.5)	225 (83.0)
Data not available, n	5	8		4	3	8	28
PR positive	49 (60.5)	45 (61.6)		32 (64.0)	10 (35.7) [#]	19 (50.0)	155 (57.4)
Data not available, n	5	8		4	3	9	29
HER2 status, n (%)							
Positive	2 (25.0)	2 (14.3)		2 (20.0)	3 (33.3)	3 (23.1)	12 (22.2)
Data not available, n	78	67		44	22	34	245
Subtype, n (%)							
Luminal A	2 (25.0)	6 (43.9)		6 (60.0)	3 (33.3)	6 (46.2)	23 (42.6)
Luminal B HER2-	3 (37.5)	3 (21.4)		1 (10.0)	0	3 (23.1)	10 (18.5)
Luminal B HER2+	2 (25.0)	1 (7.1)		0	0	2 (15.4)	5 (9.3)
HER2+	0	1 (7.1)		2 (20.0)	3 (33.3)	2 (7.7)	7 (13.0)
Triple negative	1 (12.5)	2 (21.4)		1 (10.0)	3 (33.3)	1 (7.7)	9 (16.7)
Data not available, n	78	67		44	22	34	245

SD – standard deviation; IQR – interquartile range; ER – estrogen receptor; PR – progesterone receptor, HER2 - human epidermal growth factor receptor 2

[&] p=0.02 for chi-square test for comparison of mass versus calcification alone

[§] p<0.001 for chi-square test for comparison of spiculated mass versus calcification alone

[#] p<0.001 for chi-square test for comparison of asymmetric density and density with calcification

** p=0.02 for chi-square test for comparison of calcification alone versus total

B. Large tumours (≥ 15 mm)	**Mass** n=143 (22.6 %)	**Spiculated mass** n=202 (31.9 %)	**Architectural distortion** n=6 (1.0 %)	**Asymmetric density** n=158 (24.9 %)	**Density with calcification** n=94 (7.6 %)	**Calcification alone** n=31 (4.9 %)	**Total** n=634 (100.0 %)
Age, mean (SD) years	59.8 (5.5)	60.0 (5.4)	56.7 (1.6)	60.5 (5.2)	60.3 (5.7)	61.6 (5.6)	60.2 (5.4)
Mammographic density, n (%)							
1	58 (44.3)	73 (39.0)	1 (16.7)	29 (20.6)	26 (29.9)	7 (22.6)	194 (33.3)
2	68 (51.9)	105 (56.2)	5 (83.3)	99 (70.2)	55 (63.2)	19 (61.3)	351 (60.2)
3	5 (3.8)	9 (4.8)	0 (0.0)	13 (9.2)	6 (6.9)	5 (16.1) [&*]	38 (6.5)
Data not available, n	12	15	0	17	7	0	51
Tumour diameter, median (IQR) mm	22.0 (15.0-42.0)	23.0 (15.0-55.0)	21.0 (21.0-21.0)	15.0 (15.0-62.0)	23.0 (15.0-50.0)	23.0 (15.0-37.0)	23.0 (15.0-90.0)
Histologic grade, n (%)							
1	16 (11.2)	31 (15.5)	0 (0.0)	20 (12.9)	14 (14.9)	3 (9.7)	84 (13.4)
2	61 (42.7)	109 (54.5)	4 (66.7)	87 (56.1)	39 (41.4)	8 (25.8)	308 (49.0)
3	66 (46.2)	60 (30.0)	2 (33.3)	48 (31.0)	41 (43.6)	20 (64.5) [¤#§^]	237 (37.7)**
Data not available, n	0	2	0	3	0	0	5
Lymph node status, n (%)							
Positive	75 (52.5)	114 (57.3)	4 (66.7)	103 (65.6)	56 (59.6)	20 (64.5)	372 (59.1)
Data not available, n	0	3	0	1	0	0	4
Hormonal status, n (%)							
ER positive	98 (71.0)	174 (88.3)	5 (83.3)	127 (81.4)	72 (80.9)	18 (58.1) [¤“%]	494 (80.1)
Data not available, n	5	5	0	2	5	0	17
PR positive	79 (57.7)	134 (68.4)	4 (66.7)	91 (59.5)	48 (54.6)	14 (45.2)	370 (60.6)
Data not available, n	6	6	0	5	6	0	23
HER2 status, n (%)							
Positive	1 (4.4)	12 (17.7)	1 (16.7)	12 (28.6)	6 (24.0)	4 (40.0)	36 (20.7)
Data not available, n	120	134	0	116	69	21	634
Subtype, n (%)							
Luminal A	8 (38.1)	33 (50.8)	3 (50.0)	17 (40.5)	10 (41.7)	2 (20.0)	73 (43.5)
Luminal B HER2-	5 (23.8)	12 (18.5)	1 (16.7)	9 (21.4)	6 (25.09	0	33 (19.6)
Luminal B HER2+	0	8 (12.3)	1 (16.7)	10 (23.9)	5 (20.8)	1 (10.0)	25 (14.9)
HER2+	1 (4.8)	4 (6.2)	0 (0.0)	2 (4.8)	1 (4.2)	3 (30.0)	11 (6.6)
Triple negative	7 (33.3)	8 (12.3)	1 (16.7)	4 (9.5)	2 (8.3)	4 (40.0)	26 (15.5)
Data not available, n	122	137	0	116	70	21	466

SD – standard deviation; IQR – interquartile range; ER – estrogen receptor; PR – progesterone receptor, HER2 - human epidermal growth factor receptor 2

[&] p=0.01 for chi-square test for comparison of mass versus calcification alone

[*] p=0.02 for chi-square test for comparison of spiculated mass versus calcification alone

[¤] p<0.001 for chi-square test for comparison of spiculated mass versus calcification alone

[#] p<0.001 for chi-square test for comparison of architectural distortion versus calcification alone

[§] p<0.001 for chi-square test for comparison of asymmetric density versus calcification alone

[^] p=0.04 for chi-square test for comparison of density with calcification versus calcification alone

[“] p=0.01 for chi-square test for comparison of asymmetric density versus calcification alone

[%] p=0.01 for chi-square test for comparison of density with calcification versus calcification alone

** p=0.003 for chi-square test for comparison of calcification alone versus total

C. All tumours	**Mass** n=229 (24.5 %)	**Spiculated mass** n=283 (30.3 %)	**Architectural distortion** n=6 (0.6 %)	**Asymmetric density** n=212 (22.7 %)	**Density with calcification** n=125 (13.4 %)	**Calcification alone** n=78 (8.4 %)	**Total** n=933 (100.0 %)
Age, mean (SD) years	60.6 (5.6)	60.0 (5.6)	56.7 (1.6)	60.5 (5.2)	60.4 (5.5)	59.9 (6.2)	60.3 (5.5)
Mammographic density, n (%)							
1	84 (40.9)	106 (40.9)	1 (16.7)	42 (21.9)	34 (29.3)	13 (18.1) &*	280 (32.9)
2	109 (53.2)	140 (54.1)	5 (83.3)	133 (69.3)	74 (63.8)	46 (63.9)	507 (59.7)
3	12 (5.9)	13 (5.0)	0 (0.0)	17 (8.9)	8 (6.9)	13 (18.1) &*^	63 (7.4)
Data not available, n	24	24	0	20	9	6	83
Tumour diameter, median (IQR) mm	17.0 (5.0-42.0)	19.9 (6.0-55.0)	21.0 (21.0-21.0)	20.0 (4.0-62.0)	20.0 (7.0-50.0)	12.0 (1.0-37.0)	18.0 (1.0-90.0)
Histologic grade, n (%)							
1	41 (17.9)	58 (20.6)	0 (0.0)	36 (17.3)	21 (16.8)	8 (10.5)	164 (17.7)
2	101 (44.1)	154 (54.8)	4 (66.7)	110 (52.9)	55 (44.0)	30 (39.5)	454 (49.1)
3	87 (38.0)	69 (24.6)	2 (33.3)	62 (29.8)	49 (39.2)	38 (50.0) *	307 (33.2)**
Data not available, n	0	2	0	4	0	2	8
Lymph node status, n (%)							
Positive	97 (42.9)	142 (51.3)	4 (66.7)	118 (56.2)	60 (48.4)	34 (50.0)	455 (49.6)
Data not available, n	3	6	0	2	1	4	16
Hormonal status, n (%)							
ER positive	168 (76.7)	238 (88.2)	5 (83.3)	169 (82.0)	90 (76.9)	49 (70.0) *	719 (81.0)
Data not available, n	10	13	0	6	8	8	45
PR positive	128 (58.7)	179 (66.5)	4 (66.7)	123 (60.6)	58 (50.0)	33 (47.8)	12483 (71.7)
Data not available, n	11	14	0	9	9	9	52
HER2 status, n (%)							
Positive	3 (9.7)	14 (17.1)	1 (16.7)	14 (26.9)	9 (26.5)	7 (30.4)	48 (21.1)
Data not available, n	198	283	0	160	91	55	705
Subtype, n (%)							
Luminal A	10 (34.5)	39 (49.4)	3 (50.0)	23 (44.2)	13 (19.4)	8 (34.8)	96 (43.2)
Luminal B HER2-	8 (27.6)	15 (19.0)	1 (16.7)	10 (19.2)	6 (18.2)	3 (13.0)	43 (19.4)
Luminal B HER2+	2 (6.9)	9 (11.4)	1 (16.7)	10 (19.2)	5 (15.2)	3 (13.0)	30 (13.5)
HER2+	1 (3.5)	5 (6.3)	0 (0.0)	4 (7.7)	4 (12.1)	4 (17.4)	18 (8.1)
Triple negative	8 (27.6)	11 (13.9)	1 (16.7)	5 (9.6)	5 (15.2)	5 (21.7)	35 (15.8)
Data not available, n	200	204	0	160	92	55	711

SD – standard deviation; IQR – interquartile range; ER – estrogen receptor; PR – progesterone receptor, HER2 - human epidermal growth factor receptor 2

& p=0.01 for chi-square test for comparison of mass versus calcification alone

* p<0.001 for chi-square test for comparison of spiculated mass versus calcification alone

^ p=0.04 for chi-square test for comparison of density with calcification versus calcification alone

§ p=0.002 for chi-square test for comparison of asymmetric density versus calcification alone

** p=0.003 for chi-square test for comparison of calcification alone versus total
